# Differential Influence of Components Resulting from Atmospheric-Pressure Plasma on Integrin Expression of Human HaCaT Keratinocytes

**DOI:** 10.1155/2013/761451

**Published:** 2013-06-27

**Authors:** Beate Haertel, Susanne Straßenburg, Katrin Oehmigen, Kristian Wende, Thomas von Woedtke, Ulrike Lindequist

**Affiliations:** ^1^Institute of Pharmacy, Department of Pharmaceutical Biology, University of Greifswald, Friedrich-Ludwig-Jahn-Straße 17, 17487 Greifswald, Germany; ^2^Leibniz Institute for Plasma Science and Technology e.V. (INP), Campus PlasmaMed/PlasmaVitro, Felix-Hausdorff-Straße 2, 17489 Greifswald, Germany; ^3^ZIK plasmatis, Leibniz Institute for Plasma Sciences and Technology e.V. (INP), Felix-Hausdorff-Straße 2, 17489 Greifswald, Germany

## Abstract

Adequate chronic wound healing is a major problem in medicine. A new solution might be non-thermal atmospheric-pressure plasma effectively inactivating microorganisms and influencing cells in wound healing. Plasma components as, for example, radicals can affect cells differently. HaCaT keratinocytes were treated with Dielectric Barrier Discharge plasma (DBD/air, DBD/argon), ozone or hydrogen peroxide to find the components responsible for changes in integrin expression, intracellular ROS formation or apoptosis induction. Dependent on plasma treatment time reduction of recovered cells was observed with no increase of apoptotic cells, but breakdown of mitochondrial membrane potential. DBD/air plasma increased integrins and intracellular ROS. DBD/argon caused minor changes. About 100 ppm ozone did not influence integrins. Hydrogen peroxide caused similar effects compared to DBD/air plasma. In conclusion, effects depended on working gas and exposure time to plasma. Short treatment cycles did neither change integrins nor induce apoptosis or ROS. Longer treatments changed integrins as important for influencing wound healing. Plasma effects on integrins are rather attributed to induction of other ROS than to generation of ozone. Changes of integrins by plasma may provide new solutions of improving wound healing, however, conditions are needed which allow initiating the relevant influence on integrins without being cytotoxic to cells.

## 1. Introduction

Physical plasma has been defined as a completely or partly ionized gas considered as the fourth state of matter. It is characterized by electrons, positive and negative ions, neutral atoms, and neutral or charged molecules, by its temperature, different types of radiation (e.g., UVB), and by electric fields. The generation of nonthermal plasma at atmospheric pressure with a temperature close to room temperature was the basis for treating sensitive materials as, for example, living cells. The development of different plasma sources and devices led to an explosion of research in plasma medicine. First, main focus of plasma application was the improvement of chronic infected wounds [1–3] and disinfection of surgical instruments or catheters [4] since plasma is effective in the inactivation of different microorganisms [5–10] and removal of biofilms [11–13]. Meanwhile, nonthermal plasma was also investigated for application in several other fields, for example, in dental applications, in changing surfaces of medical implants, in treating cancer or dermatological diseases, and in plastic surgery [14–16]. Ex vivo and in vivo investigations demonstrated that neither plasma-induced UV radiation nor a temperature increase or formation of radicals had a potential risk to the treated patients and volunteers [17–19].

Treating cells in culture with plasma cell detachment with loss of viability, apoptosis, and damage of DNA were observed [20–28]. The extent of observed effects depended on the plasma source (plasma jet or needle and surface or volume DBD), treatment time (plasma dose), and on the process gas (air, argon, or helium).

In wound healing, for development of cancer metastasis or for spreading of cells on the implant surfaces adhesion molecules, as integrins, cadherins, the epidermal growth factor receptor (EGFR), are of importance. These molecules are responsible for adhesion or detachment of cells, for cell signalling, cell migration, and also for growth and differentiation [29–32] and should be influenced by plasma according to the requirements. While in wound healing migration and proliferation of keratinocytes or fibroblasts should be improved, in treating cancer besides induction of apoptosis and growth arrest, inhibition of migration is requested. Aim of a successful plasma treatment is in either case killing of desired cells without harming the surrounding healthy tissue. To fulfil these criteria it is important to know which of the components generated by plasma will cause the required effect.

The purpose of this study was to elucidate how plasma influences the expression of cell surface molecules of keratinocytes (HaCaT cells) as major cell type of the skin promoting adhesion, migration, and proliferation and which of the components resulting from plasma might be responsible for the effects observed. HaCaT cells were treated with plasma generated by surface dielectric barrier discharge (surface DBD) with air or argon as process gas, with ozone and hydrogen peroxide (H_2_O_2_) as nonradical reactive oxygen species resulting from plasma. Both ozone and H_2_O_2_ can be converted into radical reactive oxygen species, for example, into the hydroxyl radical which then can further influence treated cells.

## 2. Material and Methods

### 2.1. Materials

Cell culture flasks (T75) and 60 mm diameter Petri dishes came from TPP (Trasadingen, Switzerland). RPMI 1640 with L-glutamine, the culture medium used, fetal calf serum (FCS), and sodium azide (NaN_3_) for addition to PBS buffer were purchased from Sigma (Taufkirchen/Deisenhofen, Germany). Penicillin and streptomycin and trypsin/EDTA solution were obtained from Lonza (Verviers, Belgium) and phosphate buffered saline (PBS) came from PAA (Cölbe, Germany). Hydrogen peroxide (H_2_O_2_) was purchased from Roth (Karlsruhe, Germany). Monoclonal antibodies came either from antibodies-online (Aachen, Germany) (*α*_2_-integrin (AK-7-PE, CD49b; *α*_4_-integrin (9F10-PE, CD49d), *α*_6_-integrin (GoH3-PE, CD49f); *α*_V_-integrin (NKI-M9-PE, CD51); *β*_3_-integrin (VI.PL2-FITC, CD61)), AbD Serotec (Düsseldorf, Germany) (*α*_3_-integrin (17c6-PE, CD49c), eBioscience (Frankfurt, Germany) (*β*_1_-integrin (TS2/16-FITC, CD29)), or Biozol (Eiching, Germany) (E-cadherin (67A4-PE, CD324); epidermal growth factor receptor (EGFR, ICR10-FITC)). Annexin-V-Fluos Staining Kit was provided by Roche Diagnostics (Mannheim, Germany) and the fluorescent probe 5-(and-6)-chloromethyl-2′,7′-dichlorodihydrofluorescein diacetate, acetyl ester (CM-H_2_DCFDA) by Invitrogen (Molecular Probes, Darmstadt, Germany).

### 2.2. Cell Culture

The nontumorigenic human keratinocyte cell line HaCaT originally derived from normal human adult skin was kindly provided by Professor NE Fusening (DKFZ, Heidelberg, Germany) [33]. The cells were grown in T75 flasks in RPMI 1640 with L-glutamine supplemented with 8% FCS and 1% penicillin-streptomycin solution (10,000 IU/mL penicillin; 10,000 *μ*g/mL streptomycin) (RPMI) and maintained at 37°C in a humidified atmosphere of 5% CO_2_ and 95% air. HaCaT keratinocytes were subcultured 2 times weekly. The cell line was free of mycoplasmas as tested by PCR. 

### 2.3. Treatment of HaCaT Cells with Plasma, Ozone, or a ROS Inducer

For treatment of cells with nonthermal atmospheric pressure plasma, a surface DBD plasma arrangement based on a setup described by Oehmigen et al. [34] and Haertel et al. [22] with air or argon as working gas was used. In case of using argon plasma a closed DBD arrangement was used and prior to discharge the plasma air was eliminated by gassing with argon. In all experiments at ambient air conditions, a pulsed sinusoidal voltage of 10 kV peak (20 kHz) with a 0.413/1.223 s plasma-on/plasma-off time was used. For experiments in argon atmosphere, a pulsed sinusoidal voltage of 3 kV peak (40 kHz) with a 0.413/1.223 s plasma-on/plasma-off time was used. The plasma was formed in a thin layer above the structured side which was faced towards the liquid sample [22].

Ozone was generated by a Laboratory Ozoniser (Sander GmbH, Uetze-Eltze, Germany). Ozone concentration was monitored every 6 s by an FT-IR Fourier transformed infrared spectroscopy (FT-IR) using a multicomponent FT-IR gas analyser Gasmet CR-2000 (Ansyco). Ozone concentration above the petri dish with cells in medium was adjusted to about 100 ppm, 400 ppm, 1000 ppm, or 1800 ppm. To ensure that there is only ozone above the cells an empty Petri dish first was used to eliminate air by gassing with argon. The ozone concentration was then adjusted bypassing the cells. Only thereafter cells were exposed to ozone. Negative controls were exposed to 100% oxygen.

For treatment 1 × 10^6^ cells were seeded in 60 mm diameter Petri dishes with 4 mL RPMI and cultured for 24 h at 37°C. One hour prior to treatment cell culture medium was changed. Adherent cells were then exposed for 20 s to 300 s to surface DBD plasma, for 300 s to ozone or for 24 h to 100 *μ*M H_2_O_2_. Control cells remained untreated or were treated with 100% oxygen. Treated HaCaT cells were cultured for 1 h or 24 h at 37°C. After removing the medium adherent cells were detached by subsequent treatment with PBS/EDTA (10 min) and trypsin/EDTA in Ca^2+^/Mg^2+^-free PBS (final concentration: 0.05%/0.1%; 5 min) at 37°C and centrifuged. The cell pellet was suspended in PBS supplemented with 0.1% NaN_3_ and 1% FCS (FACS-PBS) for measuring cell surface molecules, in Annexin binding buffer (ABP) for detection of apoptosis or in RPMI for measurement of intracellular ROS. Cell number was calculated using a Neubauer chamber.

### 2.4. Surface Expression of Adhesion Molecules

Surface expression of adhesion molecules on HaCaT cells was determined by flow cytometry using phycoerythrin- (PE-) or fluorescein-isothiocyanate- (FITC-) conjugated monoclonal antibodies [22]. Cells (1 × 10^5^/50 *μ*L FACS-PBS) were incubated for 30 min at 4°C with antibodies recognizing *α*_2_-, *α*_3_-, *α*_4_-, *α*_6_-, *α*_V_-, *β*_1_-, and *β*_3_-integrins, E-cadherin, and the epidermal growth factor receptor. After washing of cells 400 *μ*L FACS-PBS were added. Optimal antibody concentrations varied from 12.5 *μ*g/mL (*α*_2_-integrin) to 50 *μ*g/mL (EGFR).

The results were expressed as percentage of cells which were stained positive (mean ± SEM). For comparing the intensity of a staining, flow cytometry data were plotted in one-parameter histograms with counts at the *y*-axis (linear scale) and fluorescence intensity at the *x*-axis (4-decade logarithmic scale). The mean fluorescence intensity (MFI) of a staining, which correlates with antigen density, was expressed as arithmetic mean in log *U*.

### 2.5. Externalization of Phosphatidylserine

Externalization of phosphatidylserine as sign of apoptosis was measured 1 h and 24 h after HaCaT cells have been treated with either plasma or ozone using an Annexin-V-Fluos Staining Kit. Briefly, 1 × 10^5^ cells were incubated for 15 min in the dark at room temperature in 100 *μ*L labeling solution consisting of 2 *μ*L Annexin-V-Fluos and 2 *μ*L PI (50 *μ*g/mL) in 100 *μ*L ABP. After incubation 400 *μ*L ABP were added and apoptosis was measured within 30 min.

### 2.6. Intracellular Reactive Oxygen Species (ROS)

Intracellular ROS were detected 1 h and 24 h after the cells were exposed to plasma using the fluorescent CM-H_2_DCFDA. The dye is a derivate of DCF-DA with an additional thiol reactive chloromethyl group enhancing the binding to intracellular components, thereby prolonging cellular retention of the dye. The nonfluorescent CM-H_2_DCFDA becomes fluorescent after deacetylation by cellular esterases and cellular oxidation by ROS. Fluorescence can be detected by a flow cytometer (FL1, green fluorescence). Cells (1 × 10^5^/100 *μ*L RPMI) were incubated with 10 *μ*M CM-H_2_DCFDA in the dark for 30 min at 37°C. After washing of cells 400 *μ*L RPMI were added. 

Adhesion molecules (FL1 and FL2), Annexin/PI (FL1 and FL2), and ROS (FL1) were measured using the FACScan (BD, Heidelberg, Germany).

All results are expressed as mean ± SEM of *N* independent experiments. The number of experiments is either given in the table or figures. SigmaStat Software was used to check statistical significance (Student's *t*-test and Mann-Whitney Rank Sum Test).

## 3. Results and Discussion

### 3.1. Cell Viability and Apoptosis after Plasma Treatment

**Figure 1:**
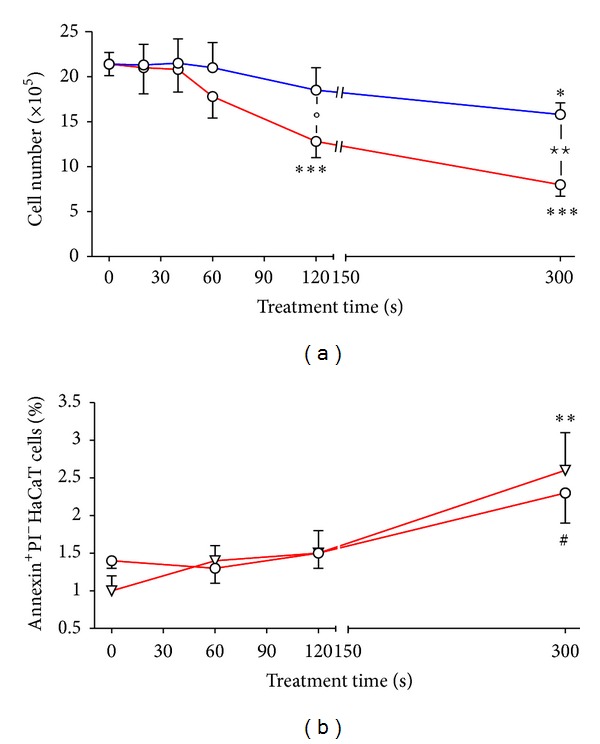
Cell number of recovered HaCaT cells (a) 24 h after treatment with surface DBD/air (red line; *N* = 8) or DBD/argon (blue line; *N* = 8) plasma for 20 s to 300 s and induction of apoptosis (b) after DBD/air treatment (▽ = 1 h after plasma treatment, ○ = 24 h after plasma treatment; *N* = 8). Apoptosis was analysed by flow cytometry using Annexin V-FITC and PI (early apoptotoc cells = Annexin V^+^PI^−^). Untreated cells served as controls (see time 0). Results are given as mean ± SEM. *P < 0.05, **P < 0.01, ***P < 0.001 (*t*-test), ^#^ *P* < 0.05 (Mann-Whitney test) versus control cells; ^⋆^ *P* < 0.05, ^⋆⋆^ *P* < 0.01 (*t*-test); °*P* < 0.05 (Mann-Whitney test).

Number of adherent cells after plasma treatment was used as sign for viability and calculated 1 h and 24 h after exposure of HaCaT cells to plasma. After 1 h (*n* = 7) neither treatment with DBD/air nor with DBD/argon changed the number of cells recovered by detachment with trypsin/EDTA (treatment time up to 300 s, data not shown). The recovery of HaCaT cells 24 h after treatment with DBD/air remained unchanged up to a treatment time of 40 s and was significantly reduced by a treatment time of 120 s. A further decrease was observed by increasing the treatment time to 300 s (Figure 1(a)). Compared to DBD/air the use of argon as working gas resulted in a milder decrease of cell number. Only the longest treatment time resulted in a significant reduced cell recovery (Figure 1(a)). Similar results for detachment of adherent cells or impaired adhesion after the treatment of different cell types with different plasma sources were reported by several investigators [20–22, 35, 36]. Viability of treated cells strongly depended on the plasma source, the treatment time/plasma dose, the working gas (e.g., air versus argon), or treatment regimen [23].

Since a loss of HaCaT cells was caused by DBD plasma treatment cell death was further characterized by staining with PI to detect DNA and Annexin V to mark extracellular phosphatidylserine residues as sign for apoptosis. Twenty-four hours after treatment, nearly all detached/unsoldered cells were positive for Annexin V independent of whether the cells remained untreated (about 85.0%) or were treated with DBD/air (>95.0%) or DBD/argon plasma (>93.0%). These cells were characterized by 25% to 35% early apoptotic cells (Annexin V positive, PI negative) and 60% to 75% late apoptotic/necrotic cells (Annexin V and PI positive; results not shown). Among the recovered adherent HaCaT cells early apoptotic cells increased slightly from 1% to about 3% (Figure 1(b)), while the amount of necrotic cells did not change significantly (data not shown). Only after a longer treatment time (20 min) apoptotic cells increase significantly (about 8% early apoptotic cells and 17% necrotic cells) [28]. Treating HaCaT cells with DBD/argon apoptosis was not induced (data not shown). Detection of apoptosis in HaCaT cells is difficult; they seem to undergo apoptosis but then they detach very quickly. First results show a breakdown of the mitochondrial membrane potential as early sign of apoptosis in recovered adherent HaCaT cells after 1 h of a 300 s treatment cycle underlying that plasma induced apoptosis in HaCaT cells. Different cell types seem to react differently to exposure of plasma. While in primary immune cells 4 h after DBD/air plasma treatment the highest amount of Annexin^+^ cells was detected [23], in primary ocular cells highest proportion of apoptotic cells was already seen 2 h after treatment [37]. In primary porcine aortic endothelial cells apoptosis was detected 24 h after treatment [38]. Apoptosis induction was further observed in primary human lymphocytes [39] and in several cancer cell lines as melanoma cells [27, 40–42], colorectal cancer cells [43, 44], lung carcinoma cells [42], or breast cancer cells [45]. It is very difficult to compare these results since different plasma sources and different methods for apoptosis detection (e.g., Annexin V staining, TUNEL assay, SubG1 phase of cell cycle, caspase 3/7, cytochrome C release) were used. Further, the time of investigation after plasma treatment differed markedly, reaching from 1 h to 72 h after treatment.

### 3.2. Plasma and Expression of Adhesion Molecules

The first target of plasma during treatment of cells is the cell membrane with its embedded proteins as, for example, proteins for ion exchange, connecting molecules like integrins or cadherins, receptors (e.g., EGFR), or several enzymes. Their expression and activity can be regulated influencing cell adhesion, migration, ion conductivity, and cell signalling. Therefore, we investigated the expression of several integrins, of E-Cadherin, and that of the EGFR 24 h after plasma exposure.

Integrins as transmembrane adhesion receptors are composed of *α*- and *β*-subunits and mediate binding of cells to components of the extracellular matrix (ECM). Nearly all HaCaT cells expressed *α*_2_-integrin (99.0% ± 0.03%, *n* = 14) and *β*_1_-integrin (98.9% ± 0.02%, *n* = 14) on their surface. This proportion was neither changed by treating the cells with DBD/air nor DBD/argon plasma (data not shown). Therefore, mean fluorescent intensity (MFI) of the integrin staining was estimated as relative indication for the amount of integrin molecules on the cell surface. 

**Figure 2:**
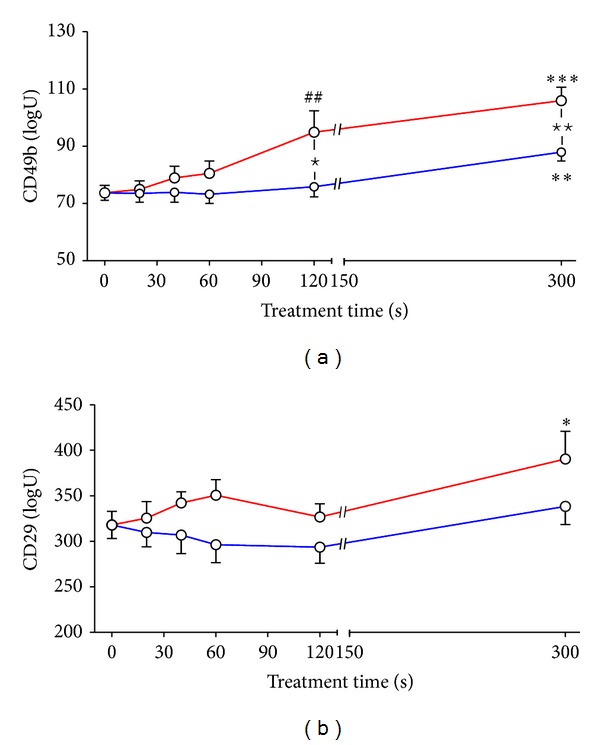
Expression of *α*_2_-integrin/CD49b (a) and *β*_1_-integrin/CD29 (b) on HaCaT cells which were recovered by trypsin 24 h after treatment with surface DBD/air (red line; *N* = 8) or DBD/argon (blue line; *N* = 7) plasma. Cells were treated for 20 s to 300 s. Untreated cells served as controls (see time 0). Expression of surface molecules was determined by flow cytometry using monoclonal antibodies. Mean fluorescence intensity (MFI) in log *U* of positive stained cells is presented (mean ± SEM). *P < 0.05, **P < 0.01, ***P < 0.001 (*t*-test); ^##^ *P* < 0.01 (Mann-Whitney test) versus control cells; ^⋆^ *P* < 0.05, ^⋆⋆^ *P* < 0.01 (*t*-test).

In control cells the MFI of *α*_2_-integrin amounted to about 70 log *U* (Figure 2(a)) which was significantly enhanced by DBD/air treatment of adherent cells for 120 s and 300 s. MFI of *β*_1_-integrin on HaCaT cells was also increased, however, by a treatment time of 300 s only (Figure 2(b)). HaCaT cells treated with DBD/argon were characterized by a slight increase of *α*_2_-integrin at 300 s treatment and a stable *β*_1_-integrin expression at all points of time tested (Figures 2(a) and 2(b)). Treating the same cells in suspension with DBD/air plasma MFI of *α*_2_-integrin decreased, while that of *β*_1_-integrin also increased [23]. The kINPen 09, a plasma jet working with argon, caused no change in *α*_2_-integrin expression on HaCaT cells treated in suspension, while that of *β*_1_-integrin increased [21]. The expression of *α*_V_- and *β*_1_-integrin on adherent fibroblasts treated with a plasma jet using helium as working gas was downregulated [24]. In human melanoma cells treated with helium plasma the expression of *α*_2_- und *α*_4_-integrin was decreased [46]. Other integrins, however, were not investigated.

**Figure 3:**
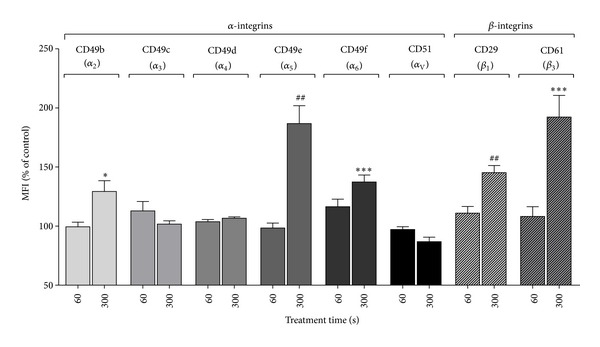
Expression of a panel of *α*- and *β*-integrins on HaCaT cells 24 h after treatment with surface DBD/air plasma (mean ± SEM, *N* = 6) for 60 s and 300 s. Expression of integrins determined by flow cytometry using monoclonal antibodies was measured as mean fluorescence intensity (MFI) of positive stained cells. The results of plasma treated cells are expressed as percentage of that of untreated control cells. *P < 0.05, ***P < 0.001 (t-test); ^##^ *P* < 0.01 (Mann-Whitney test) versus control cells.

Since we found an increase in *α*_2_-integrin expression after treating HaCaT cells with DBD/air plasma further integrins were measured 24 h after 60 s and 300 s treatment cycles. A treatment time of 60 s had no influence on the expression of all integrins investigated (Figure 3). However, in addition to *α*_2_- and *β*_1_-integrin the expression of *α*_5_-, *α*_6_-, and *β*_3_-integrins was significantly enhanced after a 300 s treatment cycle (Figure 3). In contrast, the expression of *α*_3_-, *α*_4_-, and *α*_V_-integrins was not changed by DBD/air plasma treatment for 300 s.

Different *α*-subunits can covalently bind one *β*-chain, for example, *α*_2_ *β*_1_, *α*_3_ *β*_1_, *α*_4_ *β*_1_, *α*_5_ *β*_1_, *α*_6_ *β*_1_, or *α*_V_ *β*_1_. These heterodimers bind to different components of the ECM. While the heterodimers *α*_2_ *β*_1_ and *α*_6_ *β*_1_ mediate binding to laminin, *α*_4_ *β*_1_ and *α*_5_ *β*_1_ predominantly bind to fibronectin [47]. The integrin receptor *α*_2_ *β*_1_ additionally binds to collagen. Cell spreading of HaCaT cells on fibronectin depends on, for example, the heterodimer *α*_5_ *β*_1_ integrin [48]. Especially at longer plasma treatment times, where the recovery of remaining cells is strongly decreased, expression of *α*_2_ *β*_1_, *α*_5_ *β*_1_, and *α*_6_ *β*_1_ heterodimers is enhanced, possibly to ensure adhesion of remaining cells. The integrin subunit *α*_3_ which was not influenced by plasma regulates events linked to epithelial repair including keratinocyte migration, thus promoting wound healing [49]. A loss of *α*_3_ *β*_1_ integrin would compromise intercellular adhesions and collective migration [49]. Cells which detached from the culture plate due to plasma treatment were not investigated in these experiments. However, we demonstrated a significant downregulation of *α*_2_-integrin on not-adhered HaCaT cells 24 h after treatment with the kINPen 09 while MFI of *β*_1_-integrin was not influenced ([21], Supporting Information).

Cell-cell adhesion of remaining adherent cells seems not to be influenced by DBD plasma treatment since neither the proportion of E-cadherin expressing cells nor the density of this molecule on the surface of treated HaCaT cells is changed (data not shown). Similarly also the expression of the EGFR on HaCaT cells was neither influenced by the treatment time nor by the working gas used for generation of plasma. The EGFR important for proliferation and cell growth promotes cell survival. It contributes to motility of keratinocytes, for example, in wound healing [32]. One can conclude that on recovered HaCaT cells DBD plasma does not interfere with these fundamental requirements of cell survival. Widgerow [50] reported that within a chronic wound there is a failure in expressing appropriate levels of the *α*_5_-integrin subunit on epidermal keratinocytes resulting in downregulation of *α*_5_ *β*_1_ receptors. This might contribute to the healing defect in such wounds by altered stimulation of fibronectin and keratinocytes migration. Another receptor, *α*_V_ *β*_6_, is of importance in chronic wounds. *α*_V_ *β*_6_ integrin is an epithelial cell-specific receptor and not expressed on resting epithelium, but its expression is induced in chronic wounds [51]. Widgerow [50] stated that “the activation or inhibition of integrin receptors by various agents may provide an excellent means of influencing wound healing.” DBD/air but not DBD/argon plasma increased the expression of *α*_5_ and *β*_1_ integrins significantly. Integrin *α*_V_ was decreased, however, not significantly. As demonstrated air plasma seems to be able to counteract the deleterious effects in chronic wounds, at least in terms of integrin expression.

### 3.3. Ozone and Integrin Expression

To determine whether or not ozone generated by plasma is responsible for increased integrin expression HaCaT cells were exposed to various concentrations of ozone. Prior to these experiments we measured the concentration of ozone accumulated in the gas phase during a 300 s treatment cycle (energy input about 9 J/cm^2^) with DBD/air plasma in a closed system which amounted to about 100 ppm. A different DBD plasma source caused ozone concentrations of 182 ppm (4.65 J/cm^2^) and of 30 ppm (1.95 J/cm^2^) within 15 s [52]. Exposing HaCaT keratinocytes to about 100 ppm ozone for 300 s more than 80% of adherent cells were recovered compared to the control, while after DBD/air plasma treatment for 300 s only 22.5% of cells were recovered (Table 1). In contrast to DBD/air treatment none of the integrins measured were influenced by 100 ppm ozone (Table 1). Using normal culture medium exposed to ozone and immediately applied to breast epithelial cells (MCF-10A), Kalghatgi et al. [52] could not detect any increase of *γ*-H2AX as sign for DNA damage. Increasing the ozone concentration up to 1800 ppm viability decreased continuously (Table 1). In contrast, only *α*_2_-integrin expression was similarly enhanced as after DBD/air treatment, while all other integrins investigated were not influenced (Table 1).

Taken together, although the ozone concentration with 100 ppm was 1000 times higher than the maximum allowable concentration (MAC), we could not detect any changes in the expression of those integrins which were enhanced by DBD/air plasma treatment.

### 3.4. Intracellular Reactive Oxygen Species and Integrin Expression

**Figure 4:**
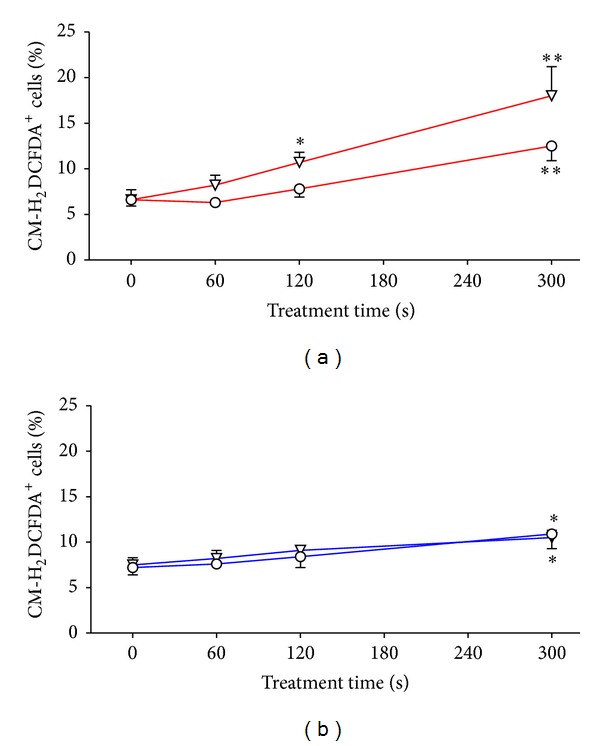
Induction of reactive oxygen species (ROS) within HaCaT cells by plasma. HaCaT cells remained either untreated (time 0) or were treated with DBD/air ((a); ▽ = 1 h after plasma treatment, ○ = 24 h after plasma treatment; *N* = 7) or DBD/argon ((b); ▽ = 1 h after plasma treatment, ○ = 24 h after plasma treatment; *N* = 7) plasma for 60 to 300 s. Intracellular ROS were detected by flow cytometry using the dye CM-H_2_DCFDA and results are given as mean ± SEM. *P < 0.05, **P < 0.01 versus control cells (*t*-test).

During plasma treatment the cells are exposed to all the electrons, ions, UV photons, short and long lived neutral and charged molecules such as ozone (O_3_), hydroxyl and superoxide radicals (HO^∙^, O_2_^∙−^), hydrogen peroxide (H_2_O_2_), nitric oxide (NO^∙^), and singlet oxygen (^1^O_2_). Reactive oxygen species always present in cells have lots of physiological functions, for example, smooth muscle relaxation, control of ventilation, signal transduction from various membrane receptors, and enhancement of immunological functions [53]. Higher concentrations of ROS lead to DNA damage and consequently to apoptosis. We wanted to know whether or not these species from plasma penetrate into the treated cells or whether plasma induces ROS intracellularly. Both mechanisms cannot be distinguished by measuring intracellular ROS using CM-H_2_DCFDA, but this method gives a general indication of the oxidation state of the cells following plasma treatment. Using this dye H_2_O_2_, peroxynitrite anion (ONOO^−^), and hydroxyl radical (HO^∙^), as well as alkylperoxyl and hydroxyl peroxyl radicals (ROO^∙^, HOO^∙^) will be detected. Treating HaCaT cells with DBD/air a linear increase of CM-H_2_DCFDA positive cells was observed 1 h after treatment (Figure 4(a)). Even 24 h later significant enhanced CM-H_2_DCFDA positive cells were still detectable (Figure 4(a)) suggesting a role for long lived radicals (e.g., O_3_, NO^∙^, H_2_O_2_) in cells induced by plasma or partly developed from short lived radicals. Changing the working gas for generating plasma from air to argon there is nearly no increase in ROS positive cells neither 1 h nor 24 h after treatment (Figure 4(b)). Taking into account that air was completely replaced by argon (prior to spark the argon plasma) and, therefore, no oxygen exists in the gas phase over the cells, the induction of intracellular ROS can only be mediated by components of the culture medium. It was hypothesized by Arjunan et al. [54] that plasma-produced ROS, as well as products from plasma-ROS interaction with cell culture medium, either diffuse through cell membrane into the cytosol or react with the cell membrane to produce intracellular ROS. Vandamme et al. [44] detected measurable reactive oxygen species in the gas phase over the cells and in the culture medium. Recently, intracellular ROS were also detected in other human cells as in primary ocular cells (keratocytes), breast cancer cells (MCF-10A), HeLa cells, and in hepatocellular carcinoma cells (HepG2) [20, 26, 37, 55]. Highest levels of intracellular ROS were found shortly after plasma treatment and reached values of untreated control cells 24 h after plasma treatment. This is very similar to that we observed.

Due to the increase in intracellular ROS especially after DBD/air plasma treatment, we assume that the increased expression of integrins (*α*_2_, *α*_5_, *α*_6_, *β*_1_, and *β*_3_) might be mediated by ROS. To verify this hypothesis HaCaT keratinocytes were treated for 24 h with H_2_O_2_. Induction of intracellular ROS was controlled and expression of those integrins which were significantly enhanced after DBD/air treatment for 300 s was measured. Hydrogen peroxide caused a nearly twofold increase of ROS positive HaCaT cells within 24 h (7.4 ± 0.8% versus 14.2 ± 1.6%, *n* = 13, *P* < 0.01), which correlated well with that caused by DBD/air treatment for 300 s (Figure 4(a)). As shown in Table 1 hydrogen peroxide decreased the number of recovered cells to about 40%. All integrins (*α*_2_, *α*_5_, *α*_6_, *β*_1_, and *β*_3_) enhanced by treating HaCaT keratinocytes with DBD/air plasma are also enhanced by exposure to hydrogen peroxide (Table 1). 

## 4. Summary and Conclusion

We provided basic investigations of HaCaT keratinocytes treated with nonthermal atmospheric-pressure plasma to get more insight into plasma-cell interaction. The first target of plasma in treating cells is the cell membrane which was influenced by DBD plasma. We demonstrated changes in the plasma membrane for phosphatidylserine residues as well as for some adhesion molecules, for example, the integrins, while E-cadherin and the EGFR were not affected. Changes in integrin expression were related to intracellular ROS induction, while ozone as nonradical ROS had no influence at concentrations measured during DBD/air treatment. Only a very high ozone concentration mediated increased *α*_2_-integrin expression.

Taken all our experiences together, we conclude that if the plasma treatment is long enough (e.g., 300 s), intracellular ROS is enhanced, and it comes to a breakdown of the mitochondrial membrane potential as early sign of apoptosis and to DNA damage [28], followed either by cell death or an increased cell surface expression of several integrins on surviving cells. Shorter treatment periods (<60 s) do neither affect the remaining cell number, intracellular ROS, nor integrin expression. Since wound related cells are not negatively influenced by a milder plasma treatment and taking into account that plasma inactivates microorganisms, one can assume that chronic infected wound healing will be improved by plasma. However, further investigations are necessary to find optimal plasma treatment conditions, which initiate the relevant influence on integrin expression, but which should have less influence on cell viability and DNA.

## Figures and Tables

**Table 1:** Cell number of HaCaT keratinocytes and integrin expression on the cell surface 24 h after exposure to different stimuli. Cells were either treated for 300 s with DBD/air plasma (6 independent experiments), DBD/argon plasma (8 independent experiments), hydrogen peroxide (6 independent experiments), or ozone (2 independent experiments), which was generated by a Laboratory Ozonizer and monitored by FT-IR. Results are expressed as % of untreated cells for plasma treatment and % of oxygen (100%) treated cells for ozone. n.d.: not determined.

	DBD		H_2_O_2_	Ozone (ppm)			
	Air	Argon	100 *μ*M	~100	~400	~1000	~1800
Cell number^1^	22.5 ± 3.0	94.9 ± 7.7	38.1 ± 3.2	83.2 ± 6.5	62.1 ± 5.2	53.4 ± 0.9	42.2 ± 1.7
*α*_ 2_-Integrin^1^	135.3 ± 4.6	117.9 ± 6.8	138.5 ± 7.8	102.5 ± 1.3	109.4 ± 18.0	115.4 ± 8.2	135.4 ± 7.7
*α*_ 5_-Integrin^1^	186.8 ± 15.1	n.d.	136.0 ± 5.7	n.d.	n.d.	n.d.	n.d.
*α*_ 6_-Integrin^1^	137.3 ± 5.9	n.d.	131.1 ± 4.8	94.7 ± 17.4	95.0 ± 8.5	79.8 ± 2.3	98.0 ± 1.2
*β*_ 1_-Integrin^1^	132.3 ± 5.1	104.8 ± 4.0	129.8 ± 5.5	98.6 ± 3.6	102.3 ± 11.9	104.5 ± 7.9	119.1 ± 2.9
*β*_ 3_-Integrin^1^	192.3 ± 18.3	n.d.	149.5 ± 13.1	n.d.	n.d.	n.d.	n.d.

^1^Mean ± SEM.

## Abbreviations

ROS: Reactive oxygen species
DBD: Dielectric Barrier Discharge
PI: Propidium iodide
ABP: Annexin binding buffer
CM-H_2_DCFDA: 5-(and-6)-Chloromethyl-2′,7′-dichlorodihydrofluorescein diacetate
EGFR: Epidermal growth factor receptor
MFI: Mean fluorescence intensity.
